# The 6-Month Efficacy of an Intensive Lifestyle Modification Program on Type 2 Diabetes Risk Among Rural Women with Prior Gestational Diabetes Mellitus: a Cluster Randomized Controlled Trial

**DOI:** 10.1007/s11121-022-01392-2

**Published:** 2022-06-30

**Authors:** Yao Chen, Qinyi Zhong, Jiaxin Luo, Yujia Tang, Mingshu Li, Qian Lin, James Allen Willey, Jyu-Lin Chen, Robin Whittemore, Jia Guo

**Affiliations:** 1 Xiangya School of Nursing, Central South University, 172 Tongzipo Road, Changsha, 410013 Hunan China; 2 Xiangya School of Public Health, Central South University, Changsha, 410078 Hinan China; 3 Philip R. Lee Institute for Health Policy Research, University of California, San Francisco, San Francisco, CA 94118 USA; 4 School of Nursing, University of California, San Francisco, San Francisco, CA 94118 USA; 5 School of Nursing, Yale University, New Haven, CT 06520 USA

**Keywords:** Woman, Gestational diabetes, Less-developed area, Prevention, Type 2 diabetes mellitus, Randomized controlled trial

## Abstract

This study aimed to evaluate the efficacy of an intensive lifestyle modification program tailored to rural Chinese women with prior gestational diabetes mellitus compared with usual care. In a cluster randomized controlled trial, 16 towns (clusters) in two distinct rural areas in China were randomly selected (8 towns per district); and 320 women with prior gestational diabetes mellitus were recruited from these towns. With stratification for the two study districts, eight towns (160 women) were randomly assigned to the intervention group of a tailored intensive lifestyle modification program and 8 towns (160 women) to the control group. Process measures were collected on attendance, engagement, fidelity, and satisfaction. Primary efficacy outcomes included glycemic and weight-related outcomes, while secondary efficacy outcomes were behavioral outcomes and type 2 diabetes risk score, which were collected at baseline, 3-month, and 6-month follow-up. Generalized estimation equations were used to analyze the data. High attendance (72% of sessions), engagement (67% of interactive activities and group discussions), fidelity (98%), and satisfaction (92%) with the tailored intensive lifestyle modification program were achieved. There were significant reductions in fasting blood glucose, oral glucose tolerance test 2 h, waist circumference, and type 2 diabetes risk score of participants in the intervention group compared to the control group (*p* < .05). There was no significant intervention effect on body mass index or behavioral outcomes (*p* > .05). In this study, we demonstrate the successful efficacy of an Intensive Lifestyle Modification Program in reducing type 2 diabetes risk among younger women with prior gestational diabetes mellitus. This tailored program delivered by local healthcare providers is a promising approach for diabetes prevention in rural China, reducing health disparities in rural communities about diabetes prevention. Registered in the Chinese Clinical Trial Registry (ChiCTR2000037956) on 3rd Jan 2018.

**Supplementary Information:**

The online version contains supplementary material available at 10.1007/s11121-022-01392-2.

## Introduction

Type 2 diabetes is a global health epidemic, causing severe complications, a high risk of premature mortality and economic burden, especially in low- and middle-income countries (Bellamy et al., 2009). Although type 2 diabetes is traditionally considered a condition of mid-to-late adulthood, early-onset type 2 diabetes (age < 40) accounts for 15–20% of total diagnosed diabetes worldwide (Sargeant et al., 2020). Diabetes prevention is rarely focused on younger or middle-aged adults (Song, 2015). Postpartum women with prior gestational diabetes mellitus (GDM) are at high risk for type 2 diabetes and are often young or middle-aged (Bellamy et al., 2009). GDM refers to any degree of glucose intolerance with onset during pregnancy (Petersmann et al., 2019). It is one of the most common pregnancy complications, with a median prevalence of 5.8–12.9% worldwide (Zhu & Zhang, 2016). Although GDM typically resolves itself after the baby is delivered, up to 40% of postpartum women with prior GDM develop a diagnosis of type 2 diabetes within 10 years (Kitzmiller et al., 2007). Considering this high risk, effective interventions are urgently needed to prevent or delay type 2 diabetes among women with prior GDM.

Lifestyle modification programs, bariatric surgery, and pharmacotherapy are efficacious treatments for diabetes prevention (Tham et al., 2014) due to the common underlying mechanism of weight loss (Lagerros & Rössner, 2013). Compared with bariatric surgery with multiple side effects and pharmacotherapy with drug dependence and untoward effects (Knowler et al., 2002; Tham et al., 2014), lifestyle modification programs have the advantages of low cost and potential for scalability without side effects and are recommended as the first-line approach worldwide (Sampson et al., 2021). Dietary and physical activity is the most common components of lifestyle interventions. Other components such as breastfeeding and psychosocial support (e.g., self-efficacy, stress management, and problem-solving) are also recommended (Sun et al., 2022). In a systematic review of lifestyle modification programs for women with prior GDM, 83% (10/12) programs utilized a combination of dietary and physical activity components, while 17% (2/12) included the dietary or physical activity component alone (Guo et al., 2016). Of that, intervention programs with a combination of multiple components (including psychosocial support) demonstrated larger effect sizes (1.11 to 5.63) than those using a single component (effect size of 0.06) for type 2 diabetes incidence. Similarly, this is often the case for insulin resistance and weight-related effect sizes.

However, the majority of intervention programs (92%) were conducted in Western countries or urban communities (Guo et al., 2016). These programs with multiple lifestyle components are complex; thus, the implementation may not be feasible or acceptable for individuals residing in less-developed economic areas. Most programs were intensively delivered in a one-on-one setting by a multi-disciplinary team of physicians, dieticians, and social workers (Tuomilehto et al., 2001). This healthcare model is not feasible in less-developed economic areas with fewer healthcare providers (Rao et al., 2011). Additionally, women in less-developed economic areas tend to be less educated, be less aware of diabetes risks, and have less healthy lifestyle behaviors than their urban counterparts (Xu et al., 2014). Therefore, lifestyle programs developed in Western countries and urban communities may not match women’s literacy level or lifestyles in less-developed economic areas (Coupe et al., 2018). Therefore, the delivery, intervention content, and dosage of the lifestyle modification programs may need to be tailored to the context of local healthcare systems and the unique needs of people in less-developed economic areas.

China has the largest population of type 2 diabetes globally (Xu et al., 2013), and the prevalence of GDM is 18.9% among pregnant Chinese women (Wei et al., 2019). There is a significant rural–urban gap in healthcare resources and population characteristics due to various historical and economic factors (Xie & Zhou, 2014). The number of urban healthcare professionals has been reported to be approximately 2.5 times that of rural healthcare professionals, even though more than half of the population (700 million people) live in rural areas (De Hert, 2020). In rural China, women with prior GDM lack adequate access to cost-effective diabetes prevention programs (Guo et al., 2018a). Tailored lifestyle modification programs are needed among this population. Such programs may also provide a template for similar programs in less-developed areas worldwide.

The aims of this study were as follows: (1) to evaluate the process measures (attendance, engagement, fidelity, and satisfaction) of a tailored intensive lifestyle modification program delivered by local healthcare providers for women with prior GDM in rural China, and (2) to examine the program’s 6-month efficacy in reducing diabetes risks compared with usual care. Primary outcomes for efficacy assessment were glycemic outcomes (fasting blood glucose [FBG] and oral glucose tolerance test 2 h [OGTT-2 h]) and weight-related outcomes (waist circumference and body mass index [BMI]). Secondary outcomes were behavioral (physical activity, dietary intake, and the intention to eat low-glycemic index [GI] foods) and type 2 diabetes risk score.

## Materials and Methods

### Study Design

A cluster randomized controlled trial was conducted to evaluate the efficacy of an intensive lifestyle modification program for rural women with prior GDM. The rural population was relatively concentrated, and towns house most rural residents in China (Zhang et al., 2020). Due to a range of social and historical factors, people with the same family name or relatively concentrated kins often reside together in the same town, and close contacts exist among these people (Zhang et al., 2020). Thus, a cluster (town) randomized controlled trial was chosen to minimize contamination between people in the same town. The trial protocol has been published (Guo et al., 2018a), and the long-term efficacy (18 months) will be reported elsewhere.

The study was conducted in two distinct rural areas in the Hunan province: Yongding County (comprises 17 towns), a county in Western Hunan with a sizeable ethnic minority population, and Youxian County (comprises 14 towns), a county in Eastern Hunan with a large ethnically Han population, to represent a different culture, lifestyle, ethnic groups in Hunan province. Eight towns from each county were randomly selected and there were no specific eligibility criteria for towns. Based on the realistic resource restriction (e.g., health system, lack of resources in town-level hospitals, and shortage of health care providers), our intervention could not be conducted concurrently in each involved town. Thus, hospital with the highest number of births in the county was chosen as the local research site in this study. The two hospitals were located in the center of the counties close to the towns.

### Participants

A convenience sample of eligible women from each town was recruited at the two hospitals from January 2018 to March 2018. The inclusion criteria for the participants were as follows: (1) women with prior GDM; (2) lived in one of the towns; (3) aged 18 or older; (4) at least 6 weeks postpartum; (5) the intention to seek primary maternal and child healthcare at the research site for at least 3 years; (6) be able to understand Mandarin Chinese. The exclusion criteria were as follows: (1) women who were pregnant or planned to be pregnant within the next 3 years; (2) a diagnosis of diabetes; and (3) other serious health problems, such as physical or cognitive disability.

A sample size of 320 women was required based on a power analysis reported in the protocol (Guo et al., 2018a, b). To improve the power of the trial, we predetermined equal cluster sizes (20 women per town) at recruitment. Once the cluster size was reached, the recruitment did not continue for the town.

### Recruitment

First, the local registered nurses who received unified training prescreened eligibility for all women who had delivered babies at the two counties via the medical records. Then, potential women were informed about the study’s purpose, benefits, and risks by phone. Registered nurses explained the study in detail and confirmed their eligibility prior to obtaining informed consent for interested women. In this study, 592 potential women from these towns were invited to participate. Of them, 272 were not interested (*n* = 215, 36.3%) or had schedule conflicts (*n* = 57, 9.6%). Of the women who were not interested in participating, 78.1% (*n* = 168) expressed that they were not interested because they did not believe they would develop diabetes.

### Randomization and Masking

A biostatistician performed the randomization via an internet randomization protocol (http://stattrek.com/statistics/random-number-generator.aspx). After recruitment, the 16 selected towns were randomly allocated to the intervention or control group in blocks to ensure that four towns in each county were assigned to them, yielding eight towns for each group with 160 women. Due to the nature of the lifestyle intervention, it is impossible to mask participants or intervention providers to the study group allocation. However, the allocation was concealed from the biostatistician in charge of developing and conducting the statistical analysis program. In addition, nurses and women were asked to sign an agreement that they would not share the treatment materials or protocol with others before completing the study.

### Interventions

Both groups received usual care based on the current clinical guidelines, including general oral information about their diabetes risk, the importance of lifestyle behavior, and a recommendation for diabetes screening every 3 years. A brochure on diabetes prevention education was provided to each participant.

The intervention group also participated in an intensive lifestyle modification program (ILSM) tailored for rural women with prior GDM. The ILSM program was adapted from the Tianjin Gestational Diabetes Mellitus Prevention Program for urban Chinese women with prior GDM (Hu et al., 2012). A participatory design involving relevant stakeholders collaborated with the research team to modify the protocol to fit the local context (Appendix I). The ILSM program included six biweekly in-person group sessions (90 min each) and eight telephone consultation sessions (20 min each) (Guo et al., 2018a). The six in-person sessions covered orientation and goal-setting, healthy eating patterns, physical activity, stress management, family support and lifestyle patterns, and farewell and relapse prevention (Table 1). Full details of the program can be obtained elsewhere (Guo et al., 2018a, b).

**Table 1:** The content of ILSM program

ILSM		
**In-person sessions**	Core sessions	Six lifestyle skills: (1) orientation and goal setting; (2) healthy eating patterns; (3) physical activity; (4) stress management; (5) family support on healthy lifestyle patterns; and (6) relapse prevention
	Frequency	60 min per session, bi-weekly
	Duration	3 months
**Maintenance sessions**	Health consultations	(1) Review of progress toward lifestyle modifications; (2) identify challenges of making change; (3) action plans; and (4) goal setting
	Format	Telephone call
	Frequency	20 review min, bi-weekly, eight calls
	Duration	3 months

Eight local registered nurses delivered the ILSM program, and each nurse was responsible for a group of 20 women from the same town. All nurses received a structured 5-day training program by the research team using the Train the Trainer Model. Training followed a standard protocol to minimize variability and maintain fidelity across all towns. The nurses were required to pass a final evaluation held by the research team, which included a scenario simulation test and a personal interview, before delivering the intervention. The ILSM protocol was strictly adhered to delivery under the supervision of trained research assistants. They attended all the in-person sessions, reminded the nurses of any missed points or excessive information, and served as a resource to the local nurses throughout the program delivery.

### Data Collection

Data collectors (i.e., research assistants and local nurses) were blinded to assignments and received unified training. The women were invited to complete self-reported questionnaires and underwent blood sample collection at the two research sites. Data for women in the two groups were collected separately to avoid contamination. The demographic and clinical data were collected at baseline. The process data on attendance, engagement, and fidelity to the ILSM program were collected throughout the program, while data on participant satisfaction were collected after the program by research assistants. Data on type 2 diabetes risk score, weight-related and lifestyle behavior outcomes were collected at baseline and the 3-month and 6-month follow-up, while glycemia were only collected at baseline and the 6-month follow-up, because it is difficult to observe changes at 3 months among people with relatively normal glycemia. Upon completing the data collection at each time point, the participants were given a small gift for their time.

### Variables and Measurements

#### Demographic, Clinical, and Implementation Data

Demographic and clinical data included participants’ age, ethnicity, education, occupation, family income, and the months after delivery. The process data included program attendance (percentage of completed in-person sessions [*n* = 6] and telephone consultations [*n* = 8]); program engagement (active participation in the five interactive activities: games, question-and-answer sessions, role-play, and group discussions—during each in-person session, for a total of 30 activities across six in-person sessions); fidelity (the extent to which nurses covered content specified in the protocol); and the overall satisfaction of participants, using a 5-point scale ranging from 5 (very satisfied) to 1 (very dissatisfied). The benchmarks for these process data were established a priori, and they included at least 70% attendance (i.e., average attendance in 10 of 14 sessions); 50% engagement in group activities (i.e., average active participation in 15 of 30 activities); 90% fidelity; and 70% satisfaction (i.e., 112 out of 160 participants selecting “very satisfied” or “satisfied” with ILSM program).

#### Primary Outcome

The biomarkers of glycemic status including *FBG* and *OGTT-2 h* data were collected via the oral 75 g glucose 2 h post-load glucose tolerance test. Blood samples were taken in the morning after an overnight fast (at least 12 h) and 2 h after the ingesting 75 g glucose. *Waist circumference* was measured on a horizontal plane, midway between the inferior margin of the ribs and the superior border of the iliac crest. *BMI* was calculated by dividing participants’ body weight in kilograms by their height in meters squared (kg/m^2^).

#### Secondary Outcome

*Physical activity* was assessed by the 8-iterm Chinese version of the International Physical Activity Questionnaire (Short version). Participants were asked the time (i.e., number of sessions and average time per session) spent on the vigorous activity, moderate-intensity activity, walking, and sitting over the last 7 days. Data were summed within each item to estimate the total time spent in physical activity per week. Then, the metabolic equivalent of the task was calculated for each participant. *Dietary intake* was assessed by a self-administered food frequency questionnaire, which asked about 36 commonly consumed staple foods, and more than 25 groups of legumes, vegetables, fruits, and dairy consumed during the past 3 months (Li et al., 2006). The questionnaire was developed based on the Dietary Guideline for Chinese Residents and modified according to local dietary patterns. *The Intention to Eat Low GI Foods* was assessed with a 24-item questionnaire using a 7-point scale, on which higher scores indicated greater intention to eat low GI foods. The Cronbach *α* ranged from 0.78 to 0.93 in previous studies and from 0.94 to 0.96 in the present study (Li et al., 2020). *Type 2 diabetes risk score* was measured by the Chinese Diabetes Risk Scale (CHINARISK) (Guo et al., 2018a), adapted from the Canadian Diabetes Risk Questionnaire. The questionnaire comprises 13 items: age, sex, BMI, waist circumference, physical activity, fruit/vegetable consumption, history of hypertension, use of antihypertension medication, history of high blood glucose, family history of diabetes, ethnicity, level of education, and history of macrosomia (birth weight over 4.0 kg). BMI and waist circumference were documented according to the measurement above, and other items were self-reported by participants. Total scores range from 0 to 88; a cutoff score of 30 or higher represents a greater 10-year risk of type 2 diabetes. Risk score has been widely used in tracking effectiveness of diabetes prevention programs over time, such as in Finland, Germany, and India (Alssema et al., 2011; Heller et al., 2020; Schmiedel et al., 2015). The sensitivity of this questionnaire is 73%, with a positive predictive value of 57% and a negative predictive value of 78%. All the measurements used in this study have been well-validated (Li et al., 2020; Marciano et al., 2019).

### Statistical Analysis

All the data were double-entered and compared for accuracy using the EpiData 3.0 software (EpiData Association, Odense, Denmark) and analyzed by SPSS (Version 22.0; Armonk, NY, USA). Descriptive statistics were used to display demographic and clinical characteristics and process measures. The demographic and clinical data of the two groups were compared using two independent samples *t*-tests and chi-square tests. In our data set, some participants missed the 3-month collection but returned at 6 months while the ANOVA for repeated measurement data required participants complete data at all time points for processing longitudinal data (Bolker et al., 2009). In addition, the time variations could affect the findings. Thus, the differences between the two groups in the efficacy outcomes from baseline to 3-month follow-up and 6-month follow-up were compared using generalized estimation equation (GEE) to indicate the effect of time, group, and group by time interaction using an intent-to-treat approach. GEE models take the correlation between repeated measurements in the same subject into account and do not require complete data for individuals at all time points (Koivusalo et al., 2016). To assess the potential effects of clusters, we calculated the intracluster correlation coefficients (ICC) for each outcome. The ICC for FBG was 0.419, and the ICCs for type 2 diabetes risk score and OGTT-2 h were 0.082 and 0.049, respectively. In general, larger ICC values are associated with larger standard errors, wider confidence intervals, and more conservative *p*-values. In order to adjust for such effects, we used fixed effects GEE regression models where the cluster itself is included as a factor within the model. A *p*-value less than 0.05 was considered statistically significant.

### Ethical Considerations

The study was approved by the Hunan Research Committee at our University in China (IRB #2,016,034) and registered on the Chinese Clinical Trial Registry (ChiCTR) (No. ChiCTR1800015023). The trial was conducted following the rules of Good Clinical Practice outlined in the Declaration of Helsinki. All participants received written and oral information about the purpose of the study from local nurses before the study. Written informed consent of all participants was obtained.

## Results

**Fig. 1:**
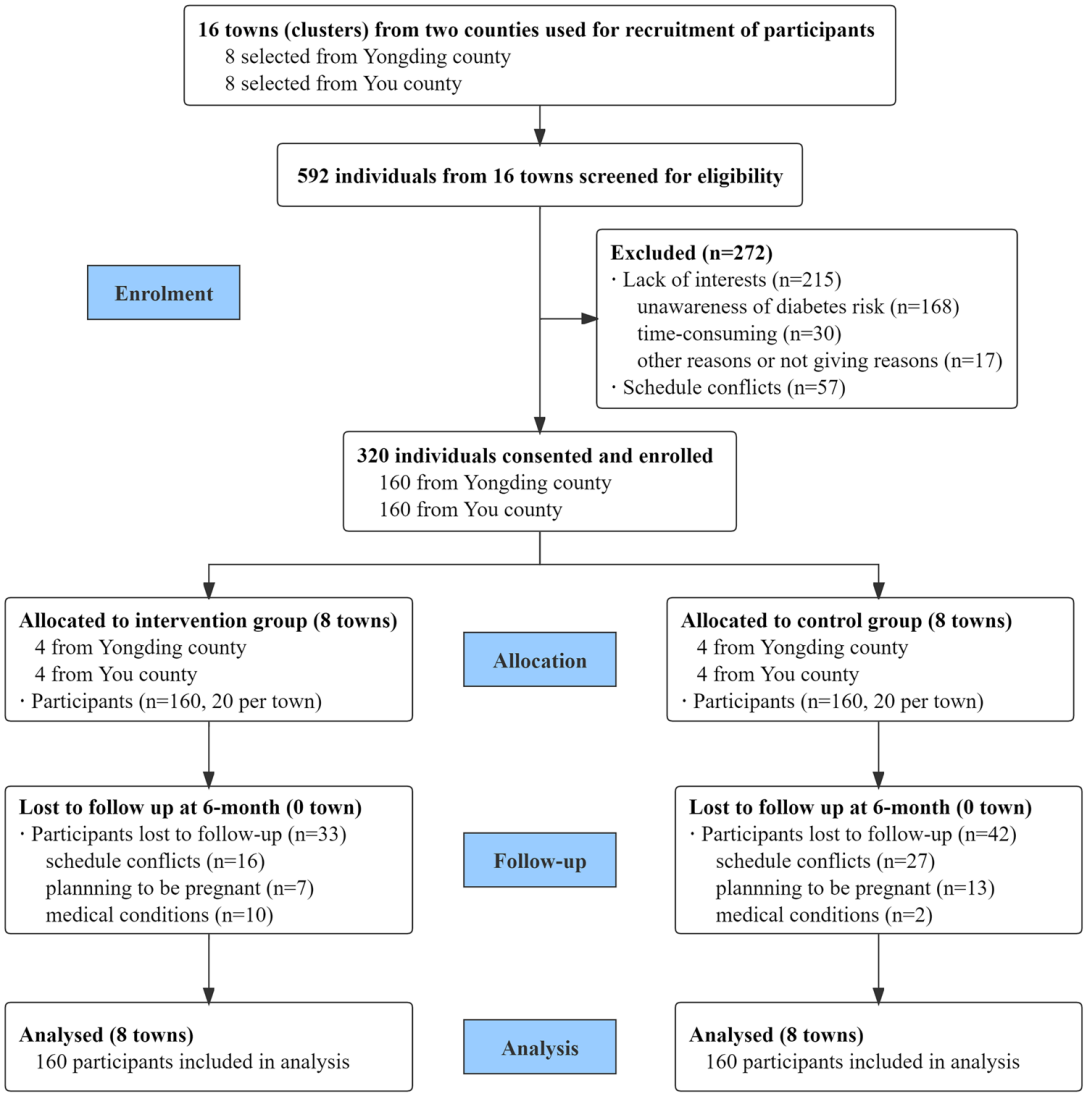
Study design and participant flow

The study sample included 320 women (54.1%) from 16 intervention (*n* = 8) and control (*n* = 8) towns who provided written informed consent; each town enrolled 20 women. At the 6-month follow-up, 245 women (127 in the intervention group and 118 in the control group) completed the data collection, with an attrition rate of 23.4%, but no town dropped out. There were no significant differences in the demographic and clinical characteristics between the 75 participants lost to follow-up and the 245 participants who completed the study (*p* > 0.05). Figure 1 summarizes the trial participants aligned with the CONSORT (Consolidated Standards for Reporting Trials) criteria for cluster randomized controlled trials (Campbell et al., 2012).

### Baseline Demographic and Clinical Characteristics

The mean age of the women was 31.92 years (*SD* = 4.91), with a range of 21–45. Most women (74.1%) reported their highest education level as senior high school or below. About one-fifth of the women (19.7%) lived with a monthly family income lower than 425 USD which considered a low family income in China (Wang et al., 2019). The mean time after delivery was 17.55 months (*SD* = 17.17). The mean BMI of the sample was 23.65 kg/m^2^ (*SD* = 3.57). There was no significant difference between the two groups for clinical or demographic characteristics (Table 2).

**Table 2:** Comparison of demographic and clinical characteristics between the intervention and the control groups at baseline

Variables	Total (*N* = 320)	ILSM group (*n* = 160)	Control group (*n* = 160)	*p*
Age, mean (SD), years	31.92 (4.91)	32.16 (5.03)	31.69 (4.80)	.386
Ethnic				.431
Han	177 (55.3%)	85 (53.1%)	92 (57.5%)	
Minority	143 (44.7%)	75 (46.9%)	68 (42.5%)	
Education				.796
Senior high school and below	237 (74.1%)	117 (73.1%)	120 (75.0%)	
College and above	83 (25.9%)	43 (26.9%)	40 (25.0%)	
Occupation				.085
Part-time job or no job	123 (38.4%)	54 (33.8%)	69 (43.1%)	
Full-time job	197 (61.6%)	106 (66.2%)	91 (56.9%)	
Family income per month				.673
< 3000 RMB ($425 USD)	63 (19.7%)	30 (18.8%)	33 (20.6%)	
≥ 3000 RMB ($425 USD)	257 (80.3%)	130 (81.3%)	127 (79.4%)	
Months after delivery, mean (SD)	17.55 (17.17)	17.38 (16.53)	17.73 (17.94)	.882
Type 2 diabetes risk score, points	24.72 (6.82)	24.77 (6.47)	24.68 (7.18)	.082
FBG, mmol/L	5.14 (0.60)	5.23 (0.61)	5.05 (0.58)	.317
OGTT-2 h, mmol/L	6.22 (1.47)	6.48 (1.44)	5.96 (1.47)	.912
BMI, kg/m^2^	23.65 (3.57)	23.75 (3.44)	23.56 (3.71)	.202
Waist circumference, cm	80.48 (8.60)	80.87 (8.17)	80.09 (9.02)	.066
Total physical activity, MET/week	47.23 (55.38)	51.02 (65.42)	43.44 (52.14)	.687
Staple food intake, g/day	360.56 (149.39)	361.65 (179.43)	359.47 (142.45)	.054
Legume intake, g/day	57.86 (77.98)	59.76 (91.92)	55.96 (56.92)	.275
Vegetable intake, g/day	180.65 (176.52)	188.97 (204.40)	172.33 (160.66)	.345
Fruit intake, g/day	190.22 (214.94)	174.41 (186.79)	206.03 (236.28)	.097
Dairy intake, mL/day	66.235 (96.01)	65.05 (92.09)	67.42 (97.56)	.496
Intention to eat low GI food, points	107.88 (20.91)	108.98 (20.67)	106.78 (21.15)	.488

Data are presented as *n* (%) or *n*/*N* (%), or mean (SD)

### Process Measures of the ILSM Program

Process data demonstrated satisfactory compliance with the program. There was a 72% attendance rate for the in-person sessions and telephone consultations of the ILSM program, with an average completion rate of 10 of the 14 sessions per woman. The engagement rate was 67%, with active participation in 20 of the 30 activities per participant on average. Program fidelity was 98%, as only one nurse did not emphasize the importance of type 2 diabetes screening every 6 months in one session (Farewell and Relapse prevention session); however, trained research assistants who attended all sessions prompted the local healthcare providers if they overlooked any content or activity; thus, the final fidelity reached 100%. The majority of the participants (92%) were very satisfied or satisfied with the ILSM program.

### Comparison of Glycemic Outcomes Between the Intervention and Control Groups from Baseline to 6-Month Follow-up

**Fig. 2:**
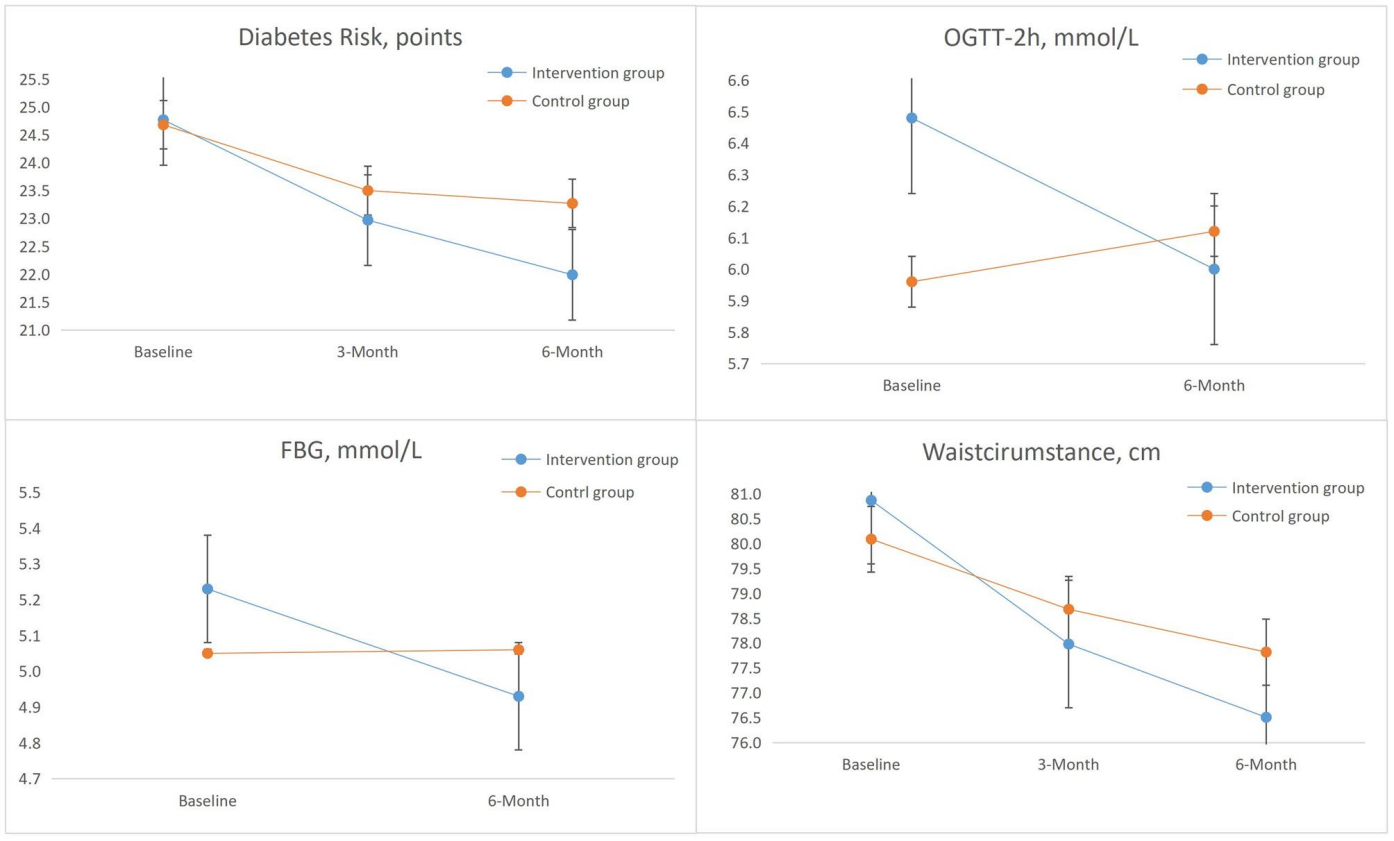
Indicated the change in FBG, OGTT-2 h, waist circumference, and T2D risk score between two groups over 6 months

There was a group by time interaction effect for FBG (*β* =  − 0.154; 95% CI =  − 0.255, − 0.053; *p* = 0.003), with a reduction of 0.30 mmol/L in the intervention group vs. 0.01 mmol/L in the control group during the 6-month study period. A favorable group by time interaction effect for OGTT-2 h was also observed (*β* =  − 0.319; 95% CI =  − 0.531, − 0.107; *p* = 0.003), with a reduction from 6.48 to 6.00 mmol/L in the intervention group, whereas the OGTT-2 h values of the control group increased by 0.16 mmol/L at the 6-month follow-up (Table 3 and Fig. 2).

**Table 3:** The 6-month efficacy of the intensive lifestyle modification program on glycemic, weight-related, and behavioral outcomes

Variables	ILSM group	Control group	*p*			ICC
			Time	Group	Group × time^a^	
FBG, mmol/L						
Baseline	5.23 (0.61)	5.05 (0.58)				
3 months	NA	NA				
6 months	4.93 (0.99)	5.06 (0.77)	**.016**	**.000**	**.003**	0.419
OGTT-2 h, mmol/L						
Baseline	6.48 (1.44)	5.96 (1.47)				
3 months	NA	NA				
6 months	6.00 (1.60)	6.12 (1.72)	**.028**	**.000**	**.003**	0.049
Waist circumference, cm						
Baseline	80.87 (8.17)	80.09 (9.02)				
3 months	77.98 (11.16)	78.68 (9.54)	.772	.227	.234	
6 months	76.51 (7.51)	77.82 (7.68)	.927	.256	**.040**	0.047
BMI, kg/m^2^						
Baseline	23.75 (3.44)	23.56 (3.71)				
3 months	22.86 (2.70)	23.16 (3.91)	.941	.304	.218	
6 months	22.37 (3.94)	21.83 (5.86)	.394	.487	.410	0.018
Total physical activity, MET/week						
Baseline	51.02 (65.42)	43.44 (52.14)				
3 months	55.08 (72.42)	48.21 (55.23)	.057	.837	.926	
6 months	58.01 (85.68)	53.61 (61.79)	.279	.395	.763	0.056
Staple food intake, g/day						
Baseline	361.65 (179.43)	359.47 (142.45)				
3 months	341.08 (159.01)	350.49 (125.88)	.853	.807	.786	
6 months	341.53 (167.85)	367.77 (202.79)	.447	.481	.354	0.073
Legume intake, g/day						
Baseline	59.76 (91.92)	55.96 (56.92)				
3 months	81.58 (78.12)	73.61 (80.90)	.951	.703	.404	
6 months	79.72 (87.46)	62.05 (74.97)	.781	.856	.306	0.069
Vegetable intake, g/day						
Baseline	188.97 (204.40)	172.33 (160.66)				
3 months	167.65 (133.28)	194.05 (247.59)	.243	.199	.147	
6 months	201.62 (188.23)	194.76 (330.31)	.175	.265	.155	0.078
Fruit intake, g/day						
Baseline	174.41 (186.79)	206.03 (236.28)				
3 months	223.25 (199.72)	191.86 (259.99)	.441	.158	.257	
6 months	201.62 (188.23)	194.98 (324.11)	.492	.156	.352	0.081
Dairy intake, mL/day						
Baseline	65.05 (92.09)	67.42 (97.56)				
3 months	81.76 (91.03)	69.61 (82.56)	.396	.307	.139	
6 months	89.52 (96.46)	67.76 (88.13)	.210	.407	.092	0.073
Intention to eat low GI food, points						
Baseline	108.98 (20.67)	106.78 (21.15)				
3 months	112.21 (17.78)	105.91 (20.82)	.075	.437	**.037**	
6 months	111.37 (17.60)	107.98 (20.15)	.224	.708	.160	0.417
Type 2 diabetes risk score, points						
Baseline	24.77 (6.47)	24.68 (7.18)				
3 months	22.97 (5.32)	23.50 (6.19)	.945	.503	.223	
6 months	21.99 (4.65)	23.27 (6.30)	.729	.428	**.016**	0.082

^a^Estimated group by time interaction effects from generalized estimation equation; *FBG*, fasting blood glucose; *OGTT-2 h*, 2-h oral glucose tolerance test; *MET*, metabolic equivalent task; *low GI*, low-glycemic index; the bolded figures indicated a significant difference

### Comparison of Weight-Related Outcomes Between the Intervention and Control Groups from Baseline to 6-Month Follow-up

A significant group by time interaction was found on waist circumference (*β* =  − 1.185; 95% CI =  − 2.317, − 0.052; *p* = 0.040), with a reduction of 4.36 cm in the intervention group vs. 2.27 cm in the control group at the 6-month follow-up. There was no significant group by time interaction on BMI between the two groups (*β* = 0.224; 95% CI =  − 0.309, 0.757; *p* = 0.410), with the BMI decreasing in both the intervention group (− 1.38) and the control group (− 1.73) after 6 months.

### Comparison of Behavioral Outcomes Between the Intervention and Control Groups from Baseline to 6-Month Follow-up

We did not find a significant group by time interaction effect for the ILSM program on total physical activity MET (*β* =  − 1.726; 95% CI =  − 12.948, 9.496; *p* = 0.763). The group by time interaction effect was not significant for any dietary outcomes, including intake of staple foods (*β* =  − 28.162; 95% CI =  − 87.764, 31.440; *p* = 0.354); legumes (*β* = 13.536; 95% CI =  − 12.390, 39.461; *p* = 0.306); vegetables (*β* =  − 55.966; 95% CI =  − 133.169, 21.237; *p* = 0.155); fruits (*β* = 38.654; 95% CI =  − 42.668, 119.976; *p* = 0.352); and dairy (*β* = 25.422; 95% CI =  − 4.136, 54.981; *p* = 0.092). A significant group by time interaction was found on the intention to eat low GI foods between the two groups at 3-month follow-up (*β* = 5.630; 95% CI = 0.342, 10.919; *p* = 0.037) but not at 6-month follow-up (*β* =  − 2.603; 95% CI =  − 6.236, 1.029; *p* = 0.160).

### Comparison of Type 2 Diabetes Risk Score Between the Intervention and Control Groups from Baseline to 6-Month Follow-up

There was a significant group by time interaction effect on type 2 diabetes risk scores (*β* =  − 1.344; 95% CI =  − 2.433, − 0.255; *p* = 0.016). As shown in Table 2, in the ILSM group, the average diabetes risk score declined from 24.77 at baseline to 21.99 at 6 months, while in the control group, the average score declined from 24.68 at baseline to 23.27 at 6 months, with a total reduction of 2.78 points vs 1.41 points.

## Discussion

The ILSM program significantly reduced glycemic status, waist circumference, and the type 2 diabetes risk score, thereby reducing the diabetes risk of rural women with prior GDM. Trained local healthcare providers implemented a tailored lifestyle modification program for rural women with prior GDM with high fidelity. The program and delivery approach can serve as a potential model for diabetes prevention that can be disseminated to other less-developed economic areas.

The ILSM program had favorable effects on glycemic status, including FBG and OGTT-2 h in women with prior GDM in rural China, which is consistent with other studies in Malaysia, Australia, and China (Hu et al., 2012; Shyam et al., 2013; Wein et al., 1999). The improved FBG and OGTT-2 h indicate better glucose regulation, which, if sustained, can contribute to a delay in diabetes progression (Ogata et al., 2018). According to a recent meta-analysis, although it is theoretically sound for lifestyle interventions to improve glycemic outcomes, quite a few programs have failed to achieve it (Gilinsky et al., 2015). An intervention design includes content, dosage, delivery, and context relevance, any of which may influence the program’s efficacy (Marciano et al., 2019). The ILSM program provided the multiple intervention content components and dosages based on scientific evidence. We retained the 12-week length of intervention, kept the components of both dietary and physical activity, and added the orientation and goal setting to improve awareness and knowledge of type 2 diabetes risk, as well as family support to increase motivation for maintaining a healthier lifestyle. We combined the recommendations of stakeholders to modify the intervention to the local context and delivered it by local healthcare providers to improve sustainability. The above details of the ILSM program may have contributed to high attendance in sessions and improvement in glycemic outcomes, which has been demonstrated in other settings (Eriksson et al., 1999).

Waist circumference was also significantly reduced after the ILSM program, which is in line with previous findings from a meta-analysis of lifestyle interventions (Hewage et al., 2020). Waist circumference is an essential indicator of abdominal obesity, which is one of the most modifiable risk factors for diabetes development among postpartum women, especially women with previous GDM (Hu et al., 2012; Wei et al., 2019). Decades of unequivocal evidence suggest that waist circumference provides both independent and additive information to BMI for predicting diabetes risk (Ross et al., 2020), and decreased waist circumference is closely associated with better insulin sensitivity (Davis et al., 2012).

The ILSM program had no significant effect on BMI reduction among women with prior GDM in this study, which contrasts with the Diabetes Prevention Studies in Finland and the USA (Eriksson et al., 1999; Ratner et al., 2008). The inconsistency may be due to the eligibility criteria for the study sample. In our study, participants were recruited with no restriction on BMI. Their mean BMI at baseline was normal (BMI < 24) with obesity rates (BMI > 28) of 12.5%, while other diabetes prevention trials only included obese participants with a mean BMI > 31 or even > 34 (Eriksson et al., 1999; Ratner et al., 2008). Additionally, the weight retention of postpartum women with gestational weight gain tends to be centrally deposited rather than peripherally deposited (Althuizen et al., 2011); thus, waist circumference may be more sensitive to change than BMI in short-term postpartum follow-up at 6 months.

There was a trend of improvement in behavioral outcomes of participants, including physical activity, diet (i.e., decreasing staple food intake, and increasing legume, fruit, and dairy intake), and the intention to eat low GI foods. However, the difference was not statistically significant at 6 months which is not in line with the results of three studies (Ferrara et al., 2016; Ratner et al., 2008; Shyam et al., 2013). First, the ILSM program aims to achieve a balanced pattern of dietary intake and physical activity, but not limited to a certain specific behavior change. However, we just assessed the consumption of specific foods instead of the comprehensive evaluation of dietary intake pattern (e.g., diet quality). In addition, the self-reported measures of physical activity used in our study may not be able to capture the change due to recall bias accurately. Participants might have misreported their physical activity by exaggerating or omitting some exercise components. Regarding physical activities, objective measures, including motion sensors and wearable devices (e.g., pedometers or accelerometers), or measures of physiological response to physical activity, such as heart rate monitors, would have been better able to do so. Second, adapting a new lifestyle pattern is a complex and gradual process affected by values, attitudes, knowledge, motivation, and actions related to individuals’ life situations (Lean et al., 2018). Thus, a 6-month follow-up may not have been long enough to allow for a significant intervention effect on specific behavior. Third, the power of the current sample size calculated for glycemic status change may be not enough to detect the change in behavioral variables.

Although there was only a trend of improving behavioral outcomes, waist circumference, FBG, and OGTT-2 h were successfully decreased. This result may suggest that the trend in improvement across multiple behaviors may be sufficient to improve physiologic measures of type 2 diabetes risk. Small changes in multiple lifestyle behaviors together may benefit weight-related outcomes and glycemic status (Lean et al., 2018). Improvement in our study may be attributed to a combined effect of goal-setting, healthy eating patterns, physical activity, stress management, family support and lifestyle patterns, and relapse prevention.

Taking the above findings together, it is not surprising that there was a significant decrease in type 2 diabetes risk score, similar to a previous study in India (Thankappan et al., 2018). With a comprehensive assessment of risk factors, type 2 diabetes risk scores are a more low-cost, noninvasive, and easy-to-administer stepwise screening approach compared with glucose makers, especially among relatively normoglycemic people (Thankappan et al., 2018). In our study, the improvement of modifiable items of the type 2 diabetes risk score measure may contribute to the significant change, including a significant reduction in waist circumference and an improvement trend in BMI, physical activity, and fruit/vegetable consumption.

This study has several limitations. First, we recruited participants with a wide range of BMI and waist circumference; thus, the efficacy of the program in specific subgroups such as women who are overweight cannot be estimated. However, the characteristics are very similar to the general population in real-world condition, especially for Asians, who tend to develop diabetes with less weight gain (Lean et al., 2018). Second, all the participants were recruited from rural areas in Hunan province; thus, the generalizability of our findings may be limited. Third, there may be a measurement error in waist circumference, as the anatomical landmarks may be difficult to locate, although the data collectors received unified training. Fourth, we did not measure the actual intake of low GI food because we lacked the specific instrument, although it was the key content in our healthy eating patterns session.

This study has several important clinical and research implications. For clinical implications, first, the ILSM program tailored for rural women in China and delivered by local healthcare providers (Train the trainer) is promising as a maternal health promotion model in areas with limited resources. However, there are challenges to reaching and retaining young women with numerous work and life responsibilities in programs that include frequent and multiple group-based sessions. Thus, the delivery format could be optimized for future dissemination to include concentrated sessions or technology-assisted content/support. Second, the ILSM program has the potential to narrow the rural–urban healthcare resource disparities about diabetes prevention.

For research implications, larger sample sizes using objective measures (e.g., pedometers) for behavioral outcomes are needed in future studies. Investigating barriers and facilitators to behavior change is essential to optimize the intervention effect and the mechanism of behavior change. Future studies on the long-term efficacy of the ILSM program and continued work on optimizing the maintenance of the short-term efficacy would be helpful. Lastly, it is essential to identify the optimum time for implementing lifestyle intervention for women with prior GDM as remission of high-risk status is less likely with longer disease transitions.

## Conclusion

The ILSM program, which was tailored for women with prior GDM in less-developed areas and delivered by local healthcare providers, was feasible, acceptable to participants, and effective in improving the glycemic status and waist circumference, thus reducing diabetes risk. Participants were also highly satisfied with the program. Future research needs to demonstrate the long-term efficacy of the ILSM program, which could then be used as a model for disseminating health promotion programs to other less-developed economic areas.

## Supplementary Information

Below is the link to the electronic supplementary material.

Supplementary file1 (DOCX 25 KB)
