# Supplementary material for: The 6-Month Efficacy of an Intensive Lifestyle Modification Program on Type 2 Diabetes Risk Among Rural Women with Prior Gestational Diabetes Mellitus: a Cluster Randomized Controlled Trial
Source: Prev Sci. 2022 Jun 30;23(7):1156–68. doi: 10.1007/s11121-022-01392-2 (PMC9489585; doi:10.1007/s11121-022-01392-2)
Supplement: Supplementary file 1 — Supplementary file1 (DOCX 25 KB) [file 11121_2022_1392_MOESM1_ESM.docx]

Our research team sequentially convened two focus groups to assess the appropriateness of components of the Tianjin-GDM-PP, identify unique needs of rural women and offer adaptation strategies in March 2017. The participants in one focus group included eight rural women with prior GDM (purposive sampling to represent different ethnicity, education level, family income, and GDM treatment regimen). All women expressed interest in the program and also offered some suggestions. They thought some components were too complicated to understand due to a lack of knowledge of T2D risk and prevention; every week of participation may be challenging due to household responsibilities and child care.

In the other focus group interview, four rural healthcare providers from our research sites (average age of 38 years old and at least five years of providing healthcare to women with GDM) and four experts in the field of nutrition, physical activities, diabetes prevention and psychology were interviewed. They suggested providing more detailed dietary guidance (such as glycemic index of common foods); teaching some easy indoor exercise (that could be done at work or home) to help positive lifestyle changes; adding two topics of stress management and family support. In addition, group-based intervention were considered to be consistent with healthcare system in rural China.

The focus group interviews were audio recorded and transcribed verbatim. Data were imported to a qualitative analysis program (Atlas.ti™) and then analyzed using an established content analysis approach (Pope & Mays, 2007). Based on the results and multiple rounds of discussion within our research team, we completed the program adaptation protocol and named it as intensive lifestyle modification (ILSM).

Subsequently, a structured delivery training, based on the Train the Trainer Model, was conducted to train rural healthcare providers how to deliver the ILSM program (Weingarten et al., 2018). In September 2017, the research team went to each research site and provided eight healthcare providers (local registered nurses, four for each research site, two sites in total) with a five full-day training. They were armed with lifestyle intervention skills (e.g. lifestyle modification protocols, intervention research, and communication) and the standard ILSM implementation procedures by reviewing the ILSM manual and practicing delivery scenarios together, etc. After that, they were asked to practice the standard procedures of each session of ILSM program one by one at the end of each day. The research team suggested some improvements according to their individual practices. Only receiving all delivery training, the local healthcare providers could be qualified as project facilitators to deliver the ILSM program.

Compared with the Tianjin Gestational Diabetes Mellitus Prevention Program for Chinese urban women with prior GDM, we contextually tailored women with prior GDM in less-developed economy areas in this study and adapted the Tianjin program. The detailed modifications are showed in the following table.

| **Session #**  **Style, and duration** | **Topics and content** | **Modifications or new activities** |
| --- | --- | --- |
| ONE:  An individualized in-person session;  With a trained rural local healthcare provider;  60 min | **T2D risk assessment and prevention**  1. Introduction to ILSM.  2. Assess personal risk of developing T2D.  3. Inform the life-threatening outcomes of having T2D.  4. Give good examples of T2D prevention by lifestyle changes.  5. Tell and ask the benefits of and barriers to lifestyle change.  6. Set goals to achieve and maintain a healthy lifestyle pattern after 3 months.  7. Group exercise (20 min). | • This new topic is added due to little knowledge on T2D risk and prevention.  • On-site indoor group exercise (20 min) is encouraged due to few exercises of the target population and the boring form of exercises.  • Child-care is served. |
| TWO:  Phone-based session;  With a trained rural local healthcare provider;  20 min | 1. Encourage each participant to describe her intention of lifestyle changes.  2. Help fix or improve some intentions.  3. Ask the barrier solutions she thinks according to Session One.  4. Schedule and encourage to the next group session. Every participant will be asked to bring a recipe of healthy dish or a cooked dish to share. | • The transcript will be added in research record for each participant.  • Motivational interviewing principles. |
| THREE:  Group session;  with a trained rural local healthcare provider;  60 min | **Healthy eating patterns**  1. Assess current eating patterns and introduce glycemic index of common food.  2. Summarize the barriers of changing unhealthy eating patterns from the previous two sessions.  3. Analyze the barrier on dietary and solutions from the previous sessions.  4. Encourage raising more barriers and solutions.  5. Help make individual action plan and coping plan on healthier eating patterns.  Action plan  • What unhealthy eating patterns would you like to change? Pattern 1, 2, 3.  • For Pattern 1, When? How?  Coping plan  • Which barrier might prevent you from changing the eating pattern? Barrier 1, 2, 3. • How could you overcome this barrier? For Barrier 1, Strategy 1.  6. Recipes sharing and home-made dishes sharing based on budget of 15 RMB per participant.  7. Group exercise (20 min) | • Participants will be asked to design a main lunch dish for the group with a budget of 15 RMB (2 dollars) one week ahead, and bring it to the group session.  • They will learn how to select healthy meals (the foods with low glycemic index) and will be involved in role-playing and practice sessions about options and choices related to high-salt, high-oil, and high-fat foods.  • Samples of healthy Chinese snacks, recipes, and information on selecting healthy food will be given to participants, especially the benefits of low-glycemic eating.  • On-site group exercise (20 min) is encouraged due to few exercises of the target population and the boring form of exercises.  • Child-care is served |
| FOUR:  Phone-based session;  With a trained rural local healthcare provider;  20 min | 1. Encourage each participant to describe her action on plans.  2. Help fix or improve some actions.  3. Ask the new barriers and provide solutions.  4. Schedule and encourage to the next group session. Every participant will be asked to bring sports shoes and clothes | • The transcript will be added in research record for each participant.  • Motivational interviewing principles. |
| FIVE:  Group session;  With an exercise physiologist and a trained rural local healthcare provider;  60 min | **Physical activities**  1. Assess current physical activities.  2. Summarize the barriers of performing physical activities.  3. Analyze the barrier’s solutions from the previous sessions.  4. Encourage raising more barriers and solutions.  5. Help make individual action plan and coping plan on physical activities.  Action plan  • Which kind of physical activity would you like to perform? Activity 1, 2, 3.  • For activity 1, When? Where? How long?  Coping plan  • Which barrier might prevent you from being active at least 2 x 20 minutes per week? Barrier 1, 2, 3.  • How could you overcome this barrier? For Barrier 1, Strategy 1.  6. Ask to perform aerobics together guiding by a popular DVD disk.  7. Group exercise (20 min) | • Women will engage in different types of noncompetitive activities (such as dance, aerobics, brisk walking and jump rope) and learn types of activities that they can do at work and at home.  • A DVD disk of aerobics will be given out.  • Child-care is served. |
| SIX:  Phone-based session;  With a trained rural local healthcare provider;  20 min | 1. Encourage each participant to describe her action on plans.  2. Help fix or improve some actions.  3. Ask the new barriers and provide solutions.  4. Schedule and encourage to the next group session. | • The transcript will be recorded.  • More focus on barriers and problem solving |
| SEVEN:  Group session;  with a psychiatrist and a trained rural local healthcare provider;  60 min | **Stress management**  1. Assess the current stress.  2. Stress management  • Impact of stress on feelings, behaviors and health  • Identify sources of stress  • Identify unhelpful stress-management strategies  • Discuss more helpful ways of managing stress  3. Help make individual action plan and coping plan on mental health management. Action plan  • Which kind of strategy would you like to perform to reduce stress? Activity 1, 2, 3.  • For activity 1, When? Where? How long?  Coping plan  • Which barrier might prevent you from managing stress? Barrier 1, 2, 3.  • How could you overcome this barrier? For Barrier 1, Strategy 1.  4. Ask to practice some psychological therapy (such as relax exercise) in groups.  5. Group exercise (20 min). | •This new topic is added due to special stress rural women experience (such as excess household responsibilities)  • Activities, stories, roleplay, lectures, DVD watching, self-consulting, and games related to dealing with stress and frustration will be used to facilitate the learning process.  • Participants will be taught to use more constructive coping and problem-solving skills to deal with stress rather than eating/drinking or playing Ma-jiang (popular recreation activity in rural areas).  • On-site group exercise (20 min) is encouraged due to few exercises of the target population and the boring form of exercises. |
| EIGHT:  Phone-based session;  With a trained rural local healthcare provider;  20 min | 1. Encourage each participant to describe her action on plans.  2. Help fix or improve some actions.  3. Ask the new barriers and provide solutions.  4. Schedule and encourage to the next group session. | • The transcript will be recorded.  • More focus on barriers and problem solving |
| NINE:  Group session;  With a trained rural local healthcare provider;  60 min | **Family support on ILSM & family lifestyle patterns.**  1. Assess the current family support on ILSM & family lifestyle patterns (meal-planning and exercise pattern) of each participant family.  2. Family healthy eating  • Healthier family meal-planning  • Dealing with barriers to making healthier choices  • Mindful eating  3. Family exercise patterns  • Importance of family exercise patterns  • Suggestions and good examples of family exercise patterns  4. Help make family action plan and coping plan with family members, on family support and family lifestyle pattern improvement.  Action plan  • Which family exercise would you like to act? Exercise pattern 1, 2, 3.  • For Pattern 1, When? How long?  Coping plan  • Which barrier might prevent you from taking the exercise pattern? Barrier 1, 2, 3.  • How could you overcome this barrier? For Barrier 1, Strategy 1.  5. Ask each family member to express their intentional support to the participant.  6. Group exercise (20 min) | •This new topic is added due to lower social economic status than urban women, especially in families, thus less family support for lifestyle modification.  • The family members are invited to practice healthy family eating planning and family physical activities together.  • On-site group exercise (20 min) is encouraged due to few exercises of the target population and the boring form of exercises.  • Child-care is served. |
| TEN:  Phone-based session;  With a trained rural local healthcare provider;  20 min | 1. Encourage each participant to describe her action on plans.  2. Help fix or improve some actions.  3. Ask the new barriers and provide solutions.  4. Schedule and encourage to the next group session. | • The transcript will be recorded.  • More focus on barriers and problem solving |
| ELEVEN:  Group session;  With a trained rural local healthcare provider;  60 min | **Fare well and Relapse prevention**  1. Assess the current lifestyle and relapse events.  2. Relapse prevention  The principle trend phases of behavior changing  Weight and energy balance  • Managing relapses  • Key strategies for maintaining a healthier lifestyle  3. Emphasize the importance of T2D screening every six months  4. Fare-well and conclusion for the group meeting sessions.  5. Group exercise (20 min). | • This topic is contained to maintain the efficacy of the previous sessions to improve lifestyle of the participants.  • On-site group exercise (20 min) is encouraged due to few exercises of the target population and the boring form of exercises.  • Child-care is served. |
| TWELVE~ FOURTEEN (Every month)  Phone-based session;  With a trained rural local healthcare provider;  ≤20 min | 1. Encourage each participant to describe her action on plans.  2. Help fix or improve some actions.  3. Ask the new barriers and provide solutions.  4. Encourage to stick to healthy lifestyle or improving to a healthier lifestyle. | • The transcript will be recorded.  • More focus on barriers and problem solving |
